# Effectiveness of Balancing Everyday Life (BEL) versus standard occupational therapy for activity engagement and functioning among people with mental illness – a cluster RCT study

**DOI:** 10.1186/s12888-017-1524-7

**Published:** 2017-11-09

**Authors:** Mona Eklund, Carina Tjörnstrand, Mikael Sandlund, Elisabeth Argentzell

**Affiliations:** 1 Department of Health Sciences/ Mental Health, Activity and Participation (MAP), Lund University, Box 157, SE-22100 Lund, Sweden; 2 Department of Clinical Science/Psychiatry, Umeå University, SE-90185 Umeå, Sweden

**Keywords:** Disability, Life style, Personal satisfaction, Occupational therapy, Recovery, Schizophrenia, Mood disorder

## Abstract

**Background:**

Many with a mental illness have an impoverished everyday life with few meaningful activities and a sedentary lifestyle. The study aim was to evaluate the effectiveness of the 16-week Balancing Everyday Life (BEL) program, compared to care as usual (CAU), for people with mental illness in specialized and community-based psychiatric services. The main outcomes concerned different aspects of subjectively evaluated everyday activities, in terms of the engagement and satisfaction they bring, balance among activities, and activity level. Secondary outcomes pertained to various facets of well-being and functioning. It was hypothesized that those who received the BEL intervention would improve more than the comparison group regarding activity, well-being and functioning outcomes.

**Methods:**

BEL is a group and activity-based lifestyle intervention. CAU entailed active support, mainly standard occupational therapy. The BEL group included 133 participants and the CAU group 93. They completed self-report questionnaires targeting activity and well-being on three occasions – at baseline, after completed intervention (at 16 weeks) and at a six-month follow-up. A research assistant rated the participants’ level of functioning and symptom severity on the same occasions. Non-parametric statistics were used since these instruments produced ordinal data.

**Results:**

The BEL group improved more than the CAU group from baseline to 16 weeks on primary outcomes in terms of activity engagement (*p* < 0.001), activity level (*p* = 0.036) and activity balance (*p* < 0.042). The BEL group also improved more on the secondary outcomes of symptom severity (*p* < 0.018) and level of functioning (*p* < 0.046) from baseline to 16 weeks, but not on well-being. High intra-class correlations (0.12–0.22) indicated clustering effects for symptom severity and level of functioning. The group differences on activity engagement (*p* = 0.001) and activity level (*p* = 0.007) remained at the follow-up. The BEL group also improved their well-being (quality of life) more than the CAU group from baseline to the follow-up (*p* = 0.049). No differences were found at that time for activity balance, level of functioning and symptom severity.

**Conclusion:**

The BEL program was effective compared to CAU in terms of activity engagement. Their improvements were not, however, greater concerning other subjective perceptions, such as satisfaction with daily activities and self-rated health, and clustering effects lowered the dependability regarding findings of improvements on symptoms and functioning. Although the CAU group had “caught up” at the follow-up, the BEL group had improved more on general quality of life. BEL appeared to be important in shortening the time required for participants to develop their engagement in activity and in attaining improved quality of life in a follow-up perspective.

**Trial registration:**

The study was registered with ClinicalTrial.gov. Reg. No. NCT02619318.

## Background

Mental illness often results in consequences such as deteriorated quality of life [1, 2], an impoverished everyday life with few meaningful activities [3], reduced work capacity [4, 5] risks of physical health problems [6] and increased mortality [7]. Most people in this situation need some form of rehabilitation, but it is not likely that one single method can address all types of consequences. For example, if the main problem is an inactive and dissatisfying everyday life in general, an activity-oriented lifestyle intervention would be appropriate [8], whereas some type of vocational training [4] would be relevant when the main problem is reduced work capacity or exclusion from the employment market. Successful programs have in recent years been developed with respect to returning to or entering the employment market [4, 9]. Interventions that address everyday life in general, and that are aimed at assisting people with mental illness in shaping a satisfying and balanced lifestyle, are less well developed. Such interventions have shown to be effective for other target groups, however, such as the Lifestyle Redesign™ to prevent ill-health among independently living older people [8] and the Redesigning Daily Occupations (ReDO)™ for people with stress-related disorders [10]. These are group-based occupational therapy programs, partly based on similar bearing principles that include mapping of the group participants’ activity history and current repertoire of everyday activities, identifying desired changes in those activities, and deciding about goals and strategies for how to accomplish changes in one’s repertoire of everyday activities [8, 11].

The Balancing Everyday Life (BEL) program [12], which was based on the same principles, was developed for people using specialized and community-based psychiatric services. The BEL program has a strong focus on accomplishing activity balance for the participants, defined as having a satisfying amount of and variation between activities [13], but also on other aspects of everyday activities, such as activity engagement [14] and valued and satisfying activities [15]. The BEL program also emphasizes personal recovery, which is defined as an individual process towards a meaningful and hopeful life, regardless of the absence or presence of symptoms [16].

Rehabilitation methods for people with enduring mental illness need to be continuously developed and improved, and standard care and traditional methods must continuously be challenged. The BEL program should thus be superior to standard psychiatric treatment in order to be regarded as an effective program.

## Methods

This was a RCT study based on cluster randomization, evaluating the effectiveness of the BEL program. The aim was to evaluate the effectiveness of the BEL program, compared to standard psychiatric treatment, for people with mental illness in specialized and community-based psychiatric services. The main outcomes concerned different aspects of subjectively evaluated everyday activities, in terms of engagement, satisfaction, balance and activity level. Secondary outcomes pertained to various facets of well-being and functioning. It was hypothesized that those who received the BEL intervention would improve more than the comparison group regarding these activity, well-being and functioning outcomes.

### Selection of settings and participants

All settings in both specialized psychiatry (outpatient units within general psychiatry and psychosis care) and community-based psychiatry (activity-based day centers) in three regions in southern and western Sweden were invited to enter the project. This entailed inclusion of settings admitting patients with a broad spectrum of disorders, such as psychoses, mood disorders and neuropsychiatric disorders. In settings that agreed to participate, a gatekeeper (an occupational therapist employed at the unit) identified clients according to the following criteria: a) self-reported imbalance between everyday activities (assessed in an interview with the gatekeeper), b) age of 18–65 years, c) substance abuse not the main diagnosis (according to team conference), d) no comorbidity of dementia or developmental disorder (according to team conference) and e) sufficient command of Swedish to participate in the data collection (assessed in an interview with the gatekeeper). All prospective participants who were eligible at the time of the project were invited and received oral and written information from the gatekeeper. Those who agreed to enter the study were then contacted by a research assistant and gave their written consent. The research assistant scheduled individual appointments, and performed the data collection in a secluded room in each setting. A total of 226 participants entered the study; 133 from BEL settings and 93 from comparison settings.

### The BEL intervention

As mentioned above the BEL was developed on the basis of previous research on lifestyle interventions made by our own group and other researchers [10, 17]. Other important sources of inspiration were descriptive studies on everyday life among people with mental illness [14, 18–20]. Clinical occupational therapists were consulted and gave their input regarding contents in the group sessions during the process of developing the first draft of the BEL. This draft was then discussed with a user panel composed of people with mental illness. They suggested fewer group sessions than originally planned and simplifications in the manual text. BEL is a group-based program (5–8 participants) consisting of 12 sessions, one session a week, and 2 booster sessions with two-week intervals. The themes for the group sessions are, e.g., activity balance, meaning and motivation, healthy living, work-related activities, leisure and relaxation, and social activities. Each session contains a brief educational section, a main group activity and a home assignment to be completed between sessions. The main group activity starts with analyzing the past and (foremost) the present situation and proceeds with identifying desired activity goals and finding strategies for how to reach them. This mapping and planning step is followed by a home assignment that means performing the desired activity in a real-life context. The home assignment is aimed at testing one of the proposed strategies. During the next group meeting, the real-life experience is evaluated and group members discuss and give each other feedback. Goals and strategies may be re-negotiated, if needed, and then the next episode that includes performing a desired activity in real life follows, a new evaluation takes place, and so forth. Self-analysis, setting goals, finding strategies and evaluating the outcome of tested strategies form a process for each session, but also for the BEL intervention as a whole. Peer support is also encouraged. The intention is that after having completed the BEL program, the participants will have developed an ability to reflect on their own situation and have gained strategies for changing their everyday life in a desired direction, such that they feel engaged in and satisfied with their everyday life and perceive a balance between rest and work, secluded and social activities, etc. The BEL program also encourages the participants to keep working with the material, preferably also together with other group members, after the group has ended in order to sustain possible new strategies in the daily life.

The BEL intervention is led by two therapists, at least one of which is an occupational therapist. In settings with only one occupational therapist, another staff member, such as a nurse or a social worker, acts as a co-therapist. As preparation, the occupational therapists take part in a specifically developed two-day education and follow the BEL manual [12]. They can then participate in a web-based discussion forum for as long as they wish where they can seek support from the researchers and/or other BEL occupational therapists. The BEL manual and materials are accessible for those who have taken the BEL education. The group leaders in the current study were generally members of a psychiatric team that could offer an array of support to patients, including psychotropic medication and counseling. The BEL intervention was provided in the premises of the psychiatric team. Fidelity to the intervention was self-rated by the occupational therapists and resulted in a median of six on a scale that ranges from one (very low fidelity) to seven (very high fidelity).

### The comparison condition

The comparison group received care as usual (CAU). The gate-keeper occupational therapists mainly invited clients they met in their clinical practice. This entailed that, although standard psychiatric treatment varied between the settings, it usually involved occupational therapy. The CAU occupational therapy sometimes included some form of group intervention, addressing for example daily living skills, social skills or creative activities, while some occupational therapists offered individual therapy only. The CAU intervention was provided by a licensed occupational therapist in all cases. The CAU therapists were part of a team that could provide a range of interventions, as was the case for those in the BEL program group. This meant that those who received CAU occupational therapy generally also received psychotropic medication, sometimes also some form of supportive therapy and/or follow-up.

### Similarity of the interventions

The standard psychiatric treatment all participants received did not differ between the BEL and the CAU interventions. Principles for “best practice” were followed and varied according to the participants’ psychiatric conditions. The additional occupational therapy treatment differed between the intervention groups, however, the BEL group receiving that intervention whereas a variation of accepted occupational therapy methods were applied for the CAU group.

### Socio-demographic and clinical data

A background questionnaire was developed to address socio-demographic factors and self-reported clinical factors, including psychiatric diagnosis or problems. The reported diagnoses/problems were then classified by a specialized psychiatrist according to the International Classification of Diseases (ICD) system [21]. This procedure was validated in a previous study, showing an expected variation in psychopathology between different diagnostic groups [22]. In addition, a number of questionnaires were used to address various aspects of activity, well-being and psychosocial functioning.

### Primary outcomes

The primary outcomes concerned various subjective ratings of everyday activities, as specified below.

#### Activity engagement

The Swedish self-rating version of the Profiles of Occupational Engagement among people with Severe mental illness (POES) [23, 24] was used. The POES consists of a diary that covers the past 24 h and has four columns; for the activity performed, the social context, the geographical context and reflections/feelings. Based on that diary, a rating is made on nine items expressing activity engagement, for example balance between rest and activity, being able to move between places, and taking initiatives. A four-point rating schedule is used. The POES has shown good psychometric properties in terms of inter-rater agreement and construct validity [23–25]. The original POES is rated by a staff member, most often an occupational therapist, but the current study was based on a self-report version. A self-report POES version limited to the productive hours of the day has shown good internal consistency, and a logical pattern of associations with occupational and functioning variables indicated construct validity [26]. No psychometric study has as yet been performed on the self-report 24-h version, but both self-report versions build on the same procedures. The internal consistency for the current sample was α = 0.85.

#### Satisfaction with daily occupations and occupational balance (SDO-OB)

To address activity satisfaction and activity balance, the Swedish version of Satisfaction with Daily Occupations and Occupational Balance (SDO-OB) assessment [27] was used. This instrument is based on the 13-item Satisfaction with Daily Occupations (SDO) scale, which has shown good psychometric proprieties when applied among people with mental illness [28]. The SDO addresses subjective perceptions of everyday activities within four domains – work, leisure, home management and self-care. Each of these domains is targeted by items with two types of questions. The first concerns whether the respondent presently performs the occupation mentioned in the item, the second about satisfaction with the occupation. The number of affirmative responses to the first type of questions forms an activity level score. The satisfaction questions are rated on a Likert-type scale ranging from 1 = worst possible to 7 = best possible satisfaction. These are summarized into a satisfaction score. The activity balance questions included in SDO-OB reflect a time allocation perspective on activity balance [29] and ask whether the individual does too little, just enough or too much within four domains – work, leisure, home management and self-care. There is also an overarching question about general activity balance. All five items use a 5-point response scale from way too little (−2) to way too much (2) to do according to their own view. The balance items from SDO-OB were recently shown to have satisfactory construct validity [27]. The balance items are analyzed separately, and do not form a scale, and only the general activity balance item was used for the present study. The satisfaction scale showed good internal consistency with the current sample, α = 0.83.

#### Activity value

The perceptions of value a person associates with his or her everyday activities was measured with the instrument Occupational Value with predefined items (OVal-pd), Swedish version [30, 31]. The participants perform a self-rating of how often they have experienced different forms of activity value in their daily life during the last month. The questions target three different forms of activity value; concrete, symbolic and self-reward value, as proposed by Persson and colleagues [32]. A revised 18-item version, which was found to form a unidimensional scale that is suitable for people with mental illness [31], was used. The participant performs a rating on a response scale from “very seldom” (=1) to “very often” (=4) regarding how often they have experienced the specific type of value. Internal consistency for the current sample was α = 0.90.

### Secondary outcomes

Secondary outcomes pertained to various measures of well-being and functioning.

#### Quality of life

The Manchester Short Assessment of Quality of Life (MANSA) [33] was used for the assessment of quality of life, here regarded as an aspect of well-being. The instrument includes 12 questions. Satisfaction with life a whole is addressed in the first question, which gives an estimate of the respondent’s general quality of life. The remaining 11 questions concern satisfaction in life areas such as employment, accommodation, social relations, mental health and physical health. The ratings from these questions, which use a seven-point scale ranging from 1  =  “could not be worse” to 7  =  “could not be better”, are summarized and reflect satisfaction with life domains. A Swedish MANSA version was used in the study. It has been psychometrically tested and has shown satisfactory properties in terms of adequate construct validity and good internal consistency [34]. Internal consistency based on the current sample was α = 0.76.

#### Self-esteem

In order to estimate self-esteem, another well-being aspect, the Rosenberg self-esteem scale was used [35]. The scale is designed to measure a global sense of self-worth and consists of ten items that cover different aspects of self-esteem, including feeling like a person of worth, on an equal plane with others. The scoring may vary, but the present study used a yes/no response format proposed by Oliver and colleagues [36]. The Rosenberg Self-esteem scale has been shown to have satisfactory item convergent and discriminant validity. It has also shown good internal consistency reliability, and to be without floor and ceiling effects [37]. A Swedish version was used, found to have good internal consistency (α = 0.90).

#### Self-rated health

Self-rated was also considered as an aspect of well-being and was assessed by using the first item of the MOS SF-36 [38]. This item targets perceived current health and a five-point scale is used, where a lower rating indicates better health. This one-item assessment has been found to be a valid indicator of subjective health [39].

#### Psychosocial functioning

The Global Assessment of Functioning (GAF) scale [40] was used to assess the individual’s overall level of psychosocial functioning. The GAF scale ranges from 0 to 100 and indicates the severity in social, psychological and occupational functioning [41, 42]. All research assistants who collected the data received training in performing the GAF rating by using videos and were calibrated against an expert GAF rater. The research assistants performed the rating at the end of the interview. Regarding psychometric testing, the GAF has demonstrated good inter-rater reliability after minimal training [43].

### Procedure for data collection

Twelve research assistants performed the data collection. Eleven of these had an occupational therapy background; three also had a Ph.D. and two were Ph.D. students. The twelfth research assistant was a final year psychology student. All of these had previous experience of working with people with mental illness. Before contacting the respondents, research assistants received training in using the instruments and received information about the stipulated procedures. These included repeating the oral and written information about the study, collecting the informed consent, the importance of administering all instruments in the same order for all participants, shaping a safe, secluded and relaxed space for the interview, and assisting the participants when needed without influencing their responses.

The participants responded to the questionnaires at the start of the BEL intervention, and after 16 weeks of intervention (including the booster sessions) the measurements were repeated. A follow-up was then made after another six months. The same data collection (instruments and procedures) was made at corresponding time points with the participants who received CAU.

### Sample size

A power calculation was based on the Satisfaction with Daily Occupations (SDO) assessment [28]. A previous study found a mean difference of 0.5 points on the SDO between groups of people with mental illness who had varying structure to their everyday life [44]. Based on the means and standard deviations from that study we arrived at 41 participants in each condition as the desired sample size to detect a difference on the SDO of 0.5 with 80% power at *p* < 0.05. Assuming a drop-out rate of 25%, we aimed to include 60 participants from each of the categories of a) BEL participants from specialized psychiatry; b) BEL participants from community-based psychiatry; c) comparison participants from specialized psychiatry; and d) comparison participants from community-based psychiatry. In line with the study aim, which does not address the influence of care context, categories a) and b) formed the BEL group and c) and d) the CAU group in this study. Comparisons between specialized and community-based psychiatry will be the topic for a related paper.

### Randomization

Cluster randomization was used to assign the settings to the BEL or the control condition. On the basis of blocks of four units, two were randomized to the BEL and two to the CAU condition. The units were included successively during 2012–2015. The randomization procedure meant that with every fourth included setting, four lots were drawn by a colleague at the department, allotting two settings to each intervention condition. This procedure ensured allocation concealment, seen as utmost important to prevent bias [45].

### Blinding

The cluster design did not allow for blinding, but other measures were taken to counteract bias. Besides allocation concealment, efforts were made to treat all participants similarly. Both groups received the same information letter, which did not indicate whether their treatment was a new method or CAU. Nor was treatment allocation specified to the research assistants who collected outcome data.

### Data analyses

The principle of intention to treat was applied, but in reality all participants who took part in the 16-week measurement had also completed the interventions. The primary analysis concerned differences in outcomes between the BEL and the CAU group and the stability of the same outcomes at the six-month follow-up. The instruments used produced ordinal scales and since equal distances between scale steps could not be assumed, mainly non-parametric statistics were used. To use the variation in an optimal way sum scores were used, and change scores were calculated as the difference between a later and a previous measurement (e.g., the follow-up score minus the baseline score). Two sets of scores were computed, namely differences from baseline to completion of the intervention and from baseline to the follow-up. The inferential statistics used to test differences in change scores between the BEL and CAU group were the Mann-Whitney U-test. For descriptive purposes, the raw change scores were transformed to T-scores; that is, all scales got the same mean (=50) and standard deviation (=10). The Wilcoxon test, performed on the respective samples separately, was used to shed further light on findings regarding change scores. In order to account for clustering effects, parametric statistics had to be used, and mixed linear model was employed to calculate intra-class correlations (ICC). The level for a statistically significant *p*-value was set at *p* < 0.05, but all *p*-values <0.1 are reported.

The software used for computations was the IBM SPSS version 23 [46].

## Results

### Settings and participants

**Fig. 1:**
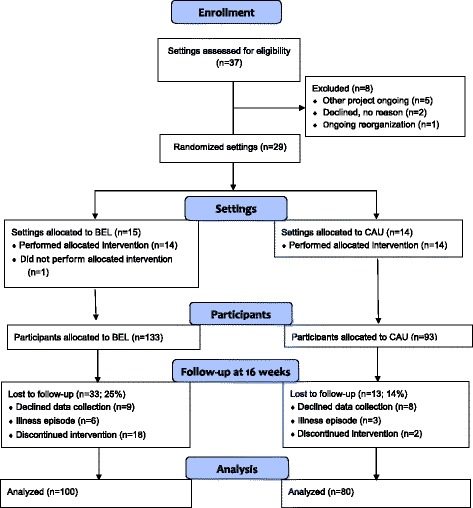
Diagram of inclusion of settings and subjects

The flow of included settings and participants is shown in Fig. 1. Out of 28 settings that were randomized to BEL or CAU, 19 came from specialized psychiatry and 9 from community-based psychiatry. Recruitment started in November 2012 and ended in March 2015, when all eligible settings in the strategically selected regions had been invited. At that time a sufficient number of participants had been recruited to the BEL intervention. Fewer than desired had been allocated to the CAU group, but the number still exceeded the minimum indicated by the power analysis. Please see Fig. 1 for further details.

The finally included participants were 133 who entered the BEL intervention and 93 who received CAU. We were unable to calculate the exact participation rate, due to dissatisfactory administrative routines in this respect, but according to the gatekeepers’ estimations the non-participants were about 20%. We also cannot present an analysis of whether these differed from the participants in any respects. According to the gatekeepers’ estimations, however, the non-participants did not differ from the participants in any noticeable respects.

The participants are described in Table 1. As seen there, females were in the majority in both groups, which were equivalent on all investigated background characteristics except for type of setting visited. A larger proportion in the BEL group than in the CAU group came from specialized psychiatry, but there were no differences regarding self-reported diagnoses.

**Table 1:** Characteristics of the participants

Characteristics	The BEL group *N* = 133	The CAU group *N* = 93	P-value
Sex (% women)	77	67	Ns. (0.094)
Age (mean, SD)	40 (11)	40 (11)	Ns.
Education (%)			Ns.
Nine-year compulsory school or lower	18	21	
High school	59	60	
College/university education	23	19	
Self-rated health (mean, SD; a lower rating denotes better health)	3.74 (0.89)	3.75 (0.96)	Ns.
Has children living at home (%)	47	47	Ns.
Has a friend (%)	83	79	Ns.
From specialized psychiatry (%)	80	59	<0.001
Self-rated diagnosis (%)			Ns.
Psychosis	19	24	
Anxiety/bipolar/depressive disorders	52	50	
ADHD/ADD	23	16	
Other	6	10	

Note. P-vales <0.10 are given

### Dropout analysis

There were 33 dropouts (25%) from baseline to 16 weeks in the BEL group and 13 (14%) in the CAU group. This was a statistically significant difference (*p* = 0.047). As shown in Fig. 1, reasons for dropping out in the BEL group mostly concerned non-compliance with the intervention. Not wanting to complete the data collection and illness episode affected both groups similarly. The dropouts did not differ from the completers on any of the variables shown in Table 1, *p*-values ranging between 0.155 and 0.712. Not shown in Fig. 1, another 11 participants (8%) in the BEL group and 10 (11%) in the CAU group dropped out between the 16-week measurement and the six-month follow-up. This was not a statistically significant difference (*p* = 0.527).

### Outcomes of the BEL intervention

**Fig. 2:**
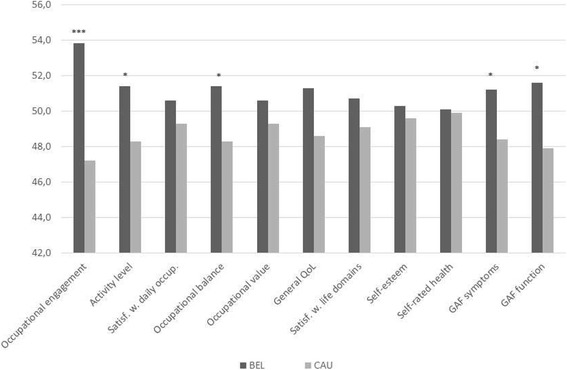
Differences between the BEL group and the CAU group on change scores (transformed to T-scores) from baseline to completed 16-wek BEL/CAU for the activity and health-related outcomes. *Note*. * p < 0.05, *** p < 0.001

Comparisons between the BEL group and the CAU group on change scores showed that the BEL group improved more in some respects. The difference in increased activity engagement was highly significant at *p* < 0.001. The other statistically significant between-group differences concerned increased activity level (*p* = 0.036), a more optimal general activity balance (*p* = 0.042), reduced symptom severity (*p* = 0.046) and increased psychosocial functioning (*p* = 0.018). The difference regarding increased general quality of life reached *p* = 0.061. Figure 2 presents change scores transformed to T-distribution.

At the follow-up (not shown in Fig. 2), the between-group group differences concerned activity engagement (*p* = 0.001), activity level (*p* = 0.007) and general quality of life (*p* = 0.049). The BEL group had improved more than the CAU group on all of these variables.

To further highlight the changes, Table 2 shows within-group changes based on scores for the BEL and CAU group separately at baseline, after 16 weeks (which corresponds to the completion of the BEL intervention) and at the six-month follow-up. As seen there, the BEL participants improved on all activity and health-related outcomes except self-rated health from baseline to completion of the intervention. Self-rated health had improved at the follow-up, however, and all other outcomes except for GAF functioning were stable in the follow-up perspective when comparing with baseline. The CAU group improved only on satisfaction with daily activities during the 16-week period that corresponded to the BEL intervention. At the follow-up, the CAU group had improved also on all other factors except for activity level compared to baseline. Comparisons between the 16-week measurement and the follow-up showed fewer statistically significant changes. Activity engagement and general quality of life improved further in the BEL group, whereas general quality of life, self-esteem and both symptoms and functioning according to GAF increased further in the CAU group.

**Table 2:** Activity and well-being factors in the two groups at baseline, after 16 weeks (BEL *N* = 100; CAU *N* = 80) and at the 6-month follow-up (BEL *N* = 89; CAU *N* = 70)

		1. Baseline; mean (SD)	2. At 16 weeks; mean (SD)	3. At 6-month follow-up; mean (SD)	P-value for change 1–2	P-value for change 1–3	P-value for change 2–3
Activity engagement	BEL	20.4 (4.8)	22.9 (4.8)	23.8 (5.2)	**<0.001**	**<0.001**	**0.006**
	CAU	21.1 (5.9)	21.5 (5.4)	22.8 (5.2)	0.943	**0.009**	**0.001**
Activity level	BEL	7.4 (2.2)	8.1 (2.1)	8.4 (2.8)	**0.004**	**0.001**	0.693
	CAU	7.6 (2.1)	7.6 (2.1)	7.5 (1.7)	0.896	0.943	0.582
Satisfaction with daily activities	BEL	63.2 (15.2)	69.1 (13.6)	71.5 (16.2)	**<0.001**	**<0.001**	0.060
	CAU	65.1 (16.7)	69.1 (15.6)	68.7 (16.9)	**0.002**	**0.02**	0.858
Activity balance ^a^	BEL	−0.6 (0.9)	−0.2 (0.8)	−0.3 (0.8)	**0.006**	**0.006**	0.786
	CAU	−0.5 (0.8)	−0.4 (0.8)	−0.4 (0.9)	0.651	0.348	0.971
Activity value	BEL	40.9 (9.3)	44 (8.9)	45.5 (11)	**0.001**	**<0.001**	0.086
	CAU	42.7 (9.7)	44.7 (9.7)	44.6 (8.1)	0.102	**0.016**	0.553
General QoL	BEL	3.3 (1.3)	3.9 (1.3)	4.2 (1.5)	**<0.001**	**<0.001**	**0.027**
	CAU	3.5 (1.5)	3.9 (1.4)	4 (1.5)	0.04	**0.008**	0.583
Satisfaction with life domains	BEL	32.1 (8.5)	34.8 (7.6)	35.8 (9.2)	**0.003**	**0.001**	0.331
	CAU	33.3 (8.3)	34.2 (7.6)	35.4 (8.8)	0.551	0.167	0.077
Self-esteem ^b^	BEL	−0.2 (0.6)	−0.1 (0.7)	−0.03 (0.7)	**0.011**	**0.009**	0.984
	CAU	−0.2 (0.6)	−0.04 (0.6)	0.1 (0.6)	0.053	**<0.001**	**<0.001**
Self-rated health ^c^	BEL	3.7 (0.9)	3.6 (1.1)	3.4 (1)	0.075	**0.012**	0.304
	CAU	3.8 (1)	3.6 (0.9)	3.4 (1)	0.136	**0.009**	0.077
GAF symptoms	BEL	51.9 (10)	54.5 (10.5)	54.8 (9.9)	**0.002**	**0.003**	0.878
	CAU	52.4 (11.3)	52.4 (9.1)	55.1 (12.2)	0.583	**0.026**	**0.018**
GAF function	BEL	50.5 (12)	55.8 (10.9)	56.8 (10.9)	**<0.001**	**0.001**	0.726
	CAU	53.9 (13.1)	54.1 (10.3)	59 (13.4)	0.214	**0.003**	**0.002**

*Note*. Significant p-values are indicated in bold

^a^ Zero indicates optimal balance, a negative value under-occupation and a positive value over-occupation

^b^ Zero indicates neutral self-esteem, a negative value negative self-esteem, and a positive value positive self-esteem

^c^ A lower value denotes better health

ICC, based on mixed model analysis, are presented in Table 3 to indicate clustering effects for the investigated outcomes. They were generally low and the highest clustering effects concerned GAF symptoms and GAF functioning.

**Table 3:** Intra-class coefficients (ICC) for clustering effects at 16 weeks (BEL N = 100; CAU N = 80) and at the 6-month follow-up (BEL N = 89; CAU N = 70)

	Baseline to 16 weeks	Baseline to 6-month follow-up	16 weeks to 6-month follow-up
Activity engagement	0.13	0	0.09
Activity level	0	0.04	0.04
Satisfaction with daily activities	0.03	0	0
Activity balance	0	0.08	0.05
Activity value	0	0	0.01
General QoL	0.02	0	0
Satisfaction with life domains	0.07	0	0
Self-esteem	0	0.05	0.04
Self-rated health	0.03	0	0.04
GAF symptoms	0.12	0.38	0.43
GAF function	0.22	0.46	0.41

## Discussion

The findings indicate that the BEL intervention was more effective than CAU in supporting certain outcomes among the participants. The greater improvements in the BEL group from baseline to 16 weeks concerned “doing” aspects such as activity engagement and activity level. Moreover, the BEL group improved more regarding level of functioning and symptomology as assessed by the research assistant. Accordingly, doing and functioning seemed to be influenced by participating in the BEL program. Compared to the CAU group, the BEL participants also changed their general activity balance in a more positive direction. This meant that their feelings of being under-occupied were reduced. But their improvements were not greater concerning other subjective perceptions, such as satisfaction with daily activities, quality of life, and self-rated health. Activity balance is generally seen as the subjective perception of a satisfactory mix of everyday activities [13, 47], but the time allocation perspective on activity balance employed in the current study may be more at the doing end of a continuum of approaches to activity balance, which may vary from literally performing a certain mix of activities to the sheer feeling of balance among activities. The fact that the BEL intervention was effective in promoting general activity balance thus appears to be in line with the overall trend that the intervention was superior to CAU for improvements in doing and functioning. These findings are on par with another activity lifestyle-based intervention, the ReDO™ program, which was developed for women with stress-related disorders. It was evaluated in a quasi-experimental study and the ReDO™ was more effective than CAU for return to work [10], but findings regarding perceptions of quality of life were inconclusive [48]. The results from the two Lifestyle Redesign© projects indicated, however, that the intervention was effective in regards to a wide array of outcomes, including quality of life [8, 17]. The follow-up measurement in the current study revealed that the BEL program also promoted quality of life. Katschnig [49] has argued that quality of life is often used as an outcome measure in psychiatric care, not least because it may be linked with personal recovery, but few interventions are actually targeted towards enhancing quality of life aspects. This makes the follow-up finding of improved quality of life for the recovery-oriented BEL group an important outcome. On the other hand, a few of the benefits indicated after 16 weeks did no longer separate the two groups. These concerned activity balance and level of functioning.

The within-group changes presented in Table 2 show that the fewer between-group differences at follow-up, compared to the 16-week measurement, were due to further improvement in the CAU group during the follow-up period. The BEL group thus gained their improvements primarily during the 16-week intervention, whereas the CAU group made their increments over a longer period, including the six-month follow-up during which they continued receiving care as long as they needed. This suggests that the BEL intervention would be time-effective compared to CAU, but with no time restriction the provision of CAU over a longer period of time could catch up and approach the outcomes of the BEL.

The influence of cluster must be considered in relation to the findings. The high ICC for symptom severity and level of functioning indicate that independence was violated. These findings are less dependable because of risk of Type I errors [50]. Some clustering effect may be seen as natural in a group intervention, however, being as the group coherence and other therapeutic factors that arise in a group are shared by its members [51, 52]. The ICC for the other outcomes thus seem to be in the realm of the expected.

### Methodological considerations

Using cluster RCT design ensures the settings are distributed unsystematically to the interventions. Stipulating strict criteria for selection of participants is another step to ensure comparable groups. Both of these strategies were followed in this study. All eligible service users at the time for the project were invited to the study, which was another measure to counteract bias in the selection of participants. We were unable to calculate the exact participation rate, however, due to use of gatekeepers and dissatisfactory administrative routines with respect to registration of non-participants. This is a limitation of this study and weakens its external validity. The design did also not allow for blinding. Methodological research has indicated that allocation concealment is more important than double blinding to prevent bias [45], however, and both allocation concealment and giving all prospective participants in both groups identical research information were additional measures to strengthen the methodology. Interviewer effect is a possible bias in non-blinded studies, but was minimized by using mainly outcome measures based on self-reports. On the other hand, the participants may have felt alleged with the intervention received. This would have influenced both groups equally, however, since CAU inferred an active therapy. Social desirability is another issue that may jeopardize the reliability of data, but since there were no rights or wrongs reflected in the measures used, social desirability would not constitute any major methodological threat. As indicated by the ICC calculations, undependability in the data seemed to concern the research assistant’s GAF ratings.

Furthermore, when calculating the sample size, the influence of clustering effects was overlooked. We therefore made a post-hoc extension of the power analysis presented in the methods section, based on our expectation of a mean of 12.5 participants from each cluster and an ICC of 0.05 [53]. This resulted in 65 participants in each group, and the number of participants thus exceeded this number for all analyses performed. The dropout rate was greater in the BEL group, which is another draw-back of this study. It may be that that the dropouts were persons for which the BEL was less suited. On the other hand, the dropout analysis did not indicate any differences on known characteristics.

Out of 28 settings that were randomized to BEL or CAU, 19 came from specialized psychiatry and 9 from community-based psychiatry. This skewness in recruitment from the two care contexts was unintentional but seems logical in relation to how Swedish psychiatric care is organized, with fewer community-based centers compared to the number of settings in specialized psychiatry. Importantly, however, psychiatric care context was a characteristic that differed between the groups and might be of relevance for estimating the effectiveness of the BEL. Therefore, the impact of care context, together with other potentially influential factors such as psychotropic medication, diagnosis, sex and socio-demographic factors, will be investigated in a forthcoming study to see if these factors play a role for the possibility for benefitting from the BEL intervention. For example, one could speculate that the potential for improvements might be smaller among participants in community-based psychiatry, who according to the Swedish organization of psychiatric care are those who have a more enduring mental illness and are not in need of acute psychiatric care. That care context might influence the outcome of the BEL intervention would thus be a rivaling hypothesis, warranting careful conclusions.

Another aspect of methodological importance is how the methods for data collection influence the study participants. Completing the POES diary gives an immediate feedback to the respondent regarding activities in his/her everyday life. This may have raised awareness in both groups regarding how they use, and can use, their time. This would have had a negligible effect for the BEL participants, considering the focus of the intervention, but may have had a booster effect for the CAU group.

## Conclusion

The BEL intervention appeared effective in comparison with CAU to promote doing, activity balance, engagement and level of functioning in the target group. This was shown regarding both self-reported and interviewer-assessed outcomes. The improvements were stable at follow-up. The intervention was barely effective for perceptions of activity satisfaction and the studied aspects of well-being, with the exception that the improvement on general quality of life from baseline to the follow-up was greater in the BEL group than in the CAU group. The CAU group had in many other respects caught up with the BEL group at the follow-up. One could say that CAU was almost as effective as the BEL if assigned considerably more time, with the exemptions of activity engagement and quality of life. In conclusion, in a 16-week perspective that corresponded to the BEL intervention, the BEL was more effective than CAU in many important respects. The findings also showed that the intervention was time-effective. These conclusions are made with some caution, however, since the difference in care context between the groups might have influenced the outcomes and high ICC were identified for a few of the outcomes.

## Abbreviations

BEL: Balancing Everyday Life
CAU: Care as usual
GAF: Global Assessment of Functioning
ICD: International Classification of Diseases
MANSA: Manchester Short Assessment of Quality of Life
MOS SF-36: Medical Outcomes Study, Short Form-36
OVal-pd.: Occupational Value with predefined items
POES: Profiles of Occupational Engagement among people with Severe mental illness
RCT: Randomized Controlled Trial
ReDO™: Redesigning Daily Occupations™
SDO: Satisfaction with Daily Occupations
SDO-OB: Satisfaction with Daily Occupations and Occupational Balance
